# Exploring drivers of women’s well-being in hospitals: mapping the landscape

**DOI:** 10.1186/s12905-024-03123-x

**Published:** 2024-06-20

**Authors:** Mitra Faghihi, Aliasghar Farshad, Nasim Salehi, Dean Whitehead, Masoud Motalebi Ghayen, Bahar Izadi, Morteza Mansourian

**Affiliations:** 1 Health Education & Promotion, Occupational Health Research Center, Iran University of Medical Sciences, Tehran, Iran; 2 Professor of Occupational Health Engineering, Occupational Health Research Center, Iran University of Medical Sciences, Tehran, Iran; 3 Health Management, Edith Cowan University, Perth, WA Australia; 4 Institute of Health and Wellbeing, Federation University, Berwick, Australia; 5 Occupational Health Research Center, Iran University of Medical Sciences, Tehran, Iran; 6 Health Promotion Research Center, Iran University of Medical Science, Tehran, Iran; 7 Associate Professor of Health Education and Promotion, Health Promotion Research Center, Iran University of Medical Sciences, Tehran, Iran

**Keywords:** Healthy hospital, Health promotion, Working women, Healthy workplace, Iran

## Abstract

**Background and purpose:**

The workplace plays a key role in impacting the health and well-being of employees at various levels, including physical, psychological, and social aspects of health. This study aims to identify the drivers of a healthy environment that promotes the well-being of women employed in hospitals.

**Materials & methods:**

This qualitative study used purposive sampling to recruit a total of 48 working women across a diverse range of participants with different job categories and socio-demographic statuses. These include clinical health (e.g., nurse, head nurse, practical nurse, supervisor, physicians); allied health (e.g., diagnostic services); public health (e.g., health promotion specialists); and administrative (e.g., hospital managers). Data was collected through semi-structured interviews and were analyzed using content analysis by creating codes, sub-themes, and themes.

**Results:**

Content analysis resulted in 31 key codes, that generated 12 sub-themes and 4 key themes. These include Advancing women’s health through collaborative leadership; a Psychologically safe environment for women; Thriving for positive social connections; and Advancing holistic health for women.

**Discussion and Conclusion:**

Hospital managers and leaders play a pivotal role in creating supportive workplaces for women. They can significantly assist in prioritizing their psychological and social health through personalized approaches tailored to women’s needs, positioning them as co-designers of their health and well-being.

## Introduction

The workplace is a pivotal setting for initiating and sustaining health promotion activities [1]. Creating healthy workplaces for mutual support can involve both top-down and bottom-up approaches [2]. Focusing on workplace health promotion goes beyond only occupational and safety measures; it also includes psycho-social programs supporting sustainable development, fostering interaction with families, and encouraging community health promotion approaches [3]. The World Health Organization (WHO) has formulated a framework and guided the implementation of a ‘healthy work environment’ [3]. In this robust evidence-based model, workplace health promotion programs are meticulously developed. The components of this model can serve as an effective tool for guiding and developing resources for health and safety promotion in the workplace [4].

Studies across various countries consistently highlight the pivotal role of women in growth and development [5]. Generally, the role of women has been neglected and considered passive, particularly at the more macro levels of planning, decision-making, and the impact they can create. However, by providing equal opportunities to women, there can be more transformative changes in the organization and society for more collaboration, brainstorming, and innovations [6, 7]. As stated in the second health promotion conference in Adelaide in 1988, women are the primary health promoters in the world. Most of them work without a salary or receive the lowest wages, indicating the attention required from policymakers and institutions for fair compensation and recognition of women’s contributions [8]. In the last few decades, women have played an increasingly significant role in society, and the number of economically active women has dramatically increased in both developing and developed countries [9]. Due to the increasing involvement of women in the workplace, studies conducted on a healthy workplace during the past decade have been criticized for lacking a gender perspective, resulting in the exclusion of women and their concerns [10]. Researchers have struggled to include women in studies, and often, gender and sexual factors are not considered when designing studies and analyzing the data [11].

Hospitals are considered one of the key settings that require the enhancement of health promotion activities, given their sensitive nature. Recognizing this parallel, the implementation of health promotion strategies in hospitals becomes imperative, aligning with the multifaceted aspects of their operational framework [10]. Although women are the primary providers of health services at various levels, they face elevated levels of occupational stress due to substantial workloads, extended working hours, and time pressure. Healthcare workers, particularly doctors and nurses may be at a greater risk of depressive disorders than the general population, due to the nature of their work and long working hours [12]. Health psychology views hospital environments as cold and anxiety-inducing for employees, patients, and their families. Women working in hospitals, particularly those in the care provision may face heightened challenges due to the demanding nature of their jobs. By incorporating the principles of environmental psychology into the design of these environments, it is possible to enhance the well-being of women in healthcare [13]. For example, the night shift, a key component of working in healthcare environments, has been identified as a significant factor contributing to breast cancer [14]. This can be clearly seen in a profession such as nursing, where nurses base their work organization on shifts in arrange to guarantee the most elevated quality of care through progression of care for patients 24 h a day [15]. Research also affirms that the assessment and monitoring of cancer risk, exposure to carcinogenic substances, and occupational diseases are predominantly explored in occupations associated with men, with an emphasis on men’s exposure to hazardous substances [16]. In addition, shift work has the potential to disrupt family and social life, leading to chronic fatigue, sleepiness, and physical symptoms. However, it frequently conflicts with the natural rhythmic timing system of ordinary individuals [17].

Given the expanding roles that women undertake in both family and society, these types of jobs generate increasingly intense conflicts between their various responsibilities. Therefore, if women encounter difficulties in balancing their work and family roles, they will experience conflicts between the two, with consequences that not only impact the individual but also affect their workplace and society as a whole in the long term and an integrated manner [4]. In addition, many musculoskeletal disorders (MSD) experienced by women in the workplace may be aggravated [18]. Within the United States, work-related injuries happen twice as regularly among nurses than among the common populace, with more than half of those being MSDs. Nurses’ ought to care for their patients ceaselessly, making nurses more powerless to musculoskeletal strains and disordes [19]. However, Work equipment, such as factory desks, chairs, and benches, is often designed to meet the ergonomic needs of an average man. Gender insensitivity in workplace design can contribute to frequent injuries among female employees, highlighting changes at the policy level for providing further support to women [18]. Creating a workplace effective in promoting women’s health involves various aspects, and addressing it requires an exploration of the understanding of work environments such as hospital settings, along with the perspectives of individuals who provide the services in these settings. Hence, this study aims to explore the drivers of promoting the health and well-being of female healthcare workers in hospital settings.

## Materials and method

This study was conducted in a public hospital in Tehran province, supported by the Iran University of Medical Sciences. The primary method of data collection was semi-structured interviews, conducted by the first author—a Ph.D. student in health education and health promotion with excellent communication skills and interview experience. Before the interviews, the researchers (interviewer) presented an introductory description of the project to potential participants to achieve an expression of interest. Following this, participants received an information sheet and consent form, and a mutually convenient time was scheduled for the interview session in the workplace of the participants (in their resting area, with only the interviewer and participant present). The interviewer maintained a neutral yet friendly relationship throughout the interviews. The participants in this study included women, from clinical health (e.g., nurse, head nurse, practical nurse, supervisor, physicians); allied health (e.g., diagnostic services); public health (e.g., health promotion specialists); and administrative (e.g., hospital managers). A voice recorder captured the data, and field notes were taken during and after each face-to-face, semi-structured interview for data collection.

The interview covered some general questions about the workplace and subsequently more specific questions were asked. There were some variations based on each case. Some examples of the questions are provided below.

- What kind of work environment do you think can improve women’s health?
- What are the dimensions and features of a workplace that promotes the health of women in hospital settings?
- What can the hospital do to enhance women’s health at work?
- What are the indicators of a health-promoting workplace for women in hospital settings?

Additional exploratory questions were tailored based on participants’ responses. Field notes and observations were integral to data collection and analysis. Each interview lasted between 20 and 30 min. To ensure accuracy, a summary of participants’ statements was presented, allowing individuals to comment and provide feedback. Some interviews were returned to participants for feedback. Data collection continued until data saturation was achieved, and no new information emerged.

In this study, the conventional content analysis method was employed to gain a deeper understanding of the phenomenon. Data collection and analysis occurred concurrently until no new codes emerged. Interviews were recorded using a voice recorder, and each interview was transcribed verbatim into a Word file. The transcripts were completed by two of the co-researchers, who cross-checked to comprehend the data and its concepts fully. Sentences and paragraphs from the interviews were treated as the smallest units of analysis and served as the primary codes. Subsequently, codes were organized into sub-categories by assessing their relevance. The final step included grouping sub-categories into key categories [20].

### Ethics approval and consent to participate

This study was approved by the Research and Ethics Council of Iran University of Medical. Sciences (code: IR.IUMS.REC.1398.201). In alignment with ethical considerations, participants were informed about the study’s purpose and necessity before interviews, and written consent was secured. To respect participants’ rights, interviews were arranged at a convenient time, and permission for audio recording was obtained. Participants were ensured confidentiality and their right to withdraw further participation if desired.

The trustworthiness and rigor of the data were assessed using Guba and Lincoln’s criteria. These include credibility, transferability, dependability, and confirmability [15, 21]. Credibility is achieved through leveraging the researcher’s long-term engagement with both the research area and participants, maintaining researchers’ impartiality, and reviewing extracted codes, sub-themes, and themes by two of the co-researchers for consensus. Transferability is achieved through rich and in-depth information achieved from participants, including numerous direct quotations and data-rich explanations. Dependability is achieved through step-by-step documentation of the data collection process and analysis technique, as well as triangulation, including seeking input from colleagues specialized in qualitative research and workplace health promotion experts. Confirmability was also done to make sure that the data collection and findings were not influenced by the researcher’s biases, using reflexivity, and member checks [22].

## Results

The majority of the participants were between 26 and 40 years old (77%), with nursing background (58%), and 48% had work experience between 2 and 10 years. The demographic information is provided in Table 1. The health-promoting environment for women working in the hospital was graphed as the central variable. Data analysis resulted in 31 codes, producing 12 sub-categories and 3 categories that is provided in Table 2. These include advancing women’s health through collaborative leadership (co-designing practical strategies, recognition through career development, empowerment through systematic networking); psychological safe environment for women’s health (fostering satisfaction, security, and joy at work, striking the balance in work-life harmony, thriving for positive social connections supporting women’s health); advancing holistic health for women (occupational health and safety, promoting healthy lifestyle behaviours, welfare of women).

**Table 1:** Demographic characteristic

		Number (percent)
Age	Less than 25	2(4.16%)
	26–30	19(39.5%)
	31–40	18(37.41)
	41–50	7(14.5)
	Above 50	2(4.16%)
Hospital work experience	2–10	23(47.91%)
	11–15	7(14.58%)
	16–20	9(18.75%)
	Above 20	9(18.75%)
Current workplace section	Men’s surgery	2(4.16%)
	CCU	7(14.58%)
	Radiology	3(6.25%)
	Dialysis	3(6.25%)
	ICU	4(8.33%)
	Education	4(8.33%)
	Internal	3(6.25%)
	Emergency	5(8.33%)
	Women’s Surgery Department	8(16.66%)
	NICU	5(8.33%)
	Administrative department	5(10.41%)
Occupations	Head nurse	7(14.58%)
	Supervisor	3(6.25%)
	Nurse	11(22.91%)
	Practical Nurse	7(14.5)
	physician	4(8.33%)
	Service staff	6(12.5%)
	Administrative department staff	5(10.41%)
	Occupational health specialist	2(4.16%)
	Health promotion specialist	3(6.25%)

**Table 2:** category, subcategory, and codes

Code	Sub-category	Categories
Facilitating policies to promote women’s health	Co-designing practical strategies	**Advancing Women’s Health through Collaborative Leadership**
Considering women’s health in hospital programs and processes		
Meritocracy	Recognition through individualized career development	
Honoring the status of women		
Human resource management according to the position of women		
Attention and provision of economic security for women working in the hospital		
Organizing teamwork in hospital departments	Empowerment through systematic networking	
Establishment of hospital work management systems		
Management style promoting women’s health		
Attention to the satisfaction and psychological security of women	Fostering satisfaction, security, and joy at work	**Psychological safe environment for women’s health**
Improving the desirability of the hospital work environment		
Creating conditions to damping women’s job burnout		
Designing programs to improve the morale of women working in the hospital		
The hospital’s support for the family conditions of working women	Striking the balance in work-life harmony	
Empowering women in adjusting work-life interference		
Planning and development to promote positive interaction between employees	Fostering solidarity and social cohesion	**Thriving for positive social connections supporting women’s health**
Developing positive working relationships between managers and employees		
Development of social support of colleagues		
Preventing managers from horizontal violence against women working in the hospital	Socially secure environment for women	
Attention to the prevention of violence against women by patients and companions		
Social security for working women in communication with colleagues		
Paying attention to ergonomics suitable for women’s conditions	Occupational health and safety	**Advancing holistic health for women**
Planning and ensuring the safety of the hospital work environment according to the characteristics of women		
Providing a healthy workplace for women in hospital conditions		
Providing the conditions and specializing in periodic examinations of women	Promoting healthy lifestyle behaviours	
Planning and developing physical activity according to the working and family conditions of working women		
Attention to healthy nutrition and healthy food for working women		
Providing childcare facilities for women working in the hospital	Welfare of women	
Attention to the welfare and health of working women and their families		
Provision of comfort facilities outside the hospital for women		

### Advancing women’s health through collaborative leadership

Women’s health through collaborative leadership is a fundamental category, indicating the importance of including women across various levels of strategies and decision-making, ensuring that they have representation and their voices are heard. This theme includes three key sub-categories: co-designing practical strategies, recognition through career development, and empowerment through systematic networking. All these sub-themes emphasize enhancing the value and recognition of women in matters related to them, rather than making decisions for them.

### Co-designing practical strategies

Initiating and/or adjusting existing policies are fundamental steps in impacting women’s health in workplaces, such as hospitals. The first step is to include women’s representatives from various relevant departments and fields in decision-making about policies and regulations. To adopt policies and decisions that are relevant and positively impact women’s health, women should play a proactive role in the decision-making process. Different approaches should be considered to attract their participation, as highlighted by the majority of women who participated in the study. Secondly, decisions need to be based on evidence-based surveys with other women to understand their needs, priorities, and preferences. For example, having women’s representatives and advocates in health promotion decision-making committees can be very beneficial in addressing the needs and priorities of women’s health and creating a healthy workplace atmosphere

There’s a manager who, while sitting in a closed-door meeting and unaccompanied by a nurse, declares a ban on cell phones. Meanwhile, a mother, who is also a nurse, is not present. In such a situation, if her child is alone at home and needs to reach out, the manager’s strict prohibition on cell phones becomes a challenge, leaving the mother in a difficult position, unaware of her child’s situation….

If women in this system do not have a representative and are not present in the meetings where decisions are being made for them, or if they are not represented, how can the right decision be made? (45-year-old lady with 18 years of experience in charge of hospital quality improvement)

”At least we should set internal regulations in the organization that is, if an organization is supporting women’s health, it is expected to develop internal regulations within the authority of the head of the organization, and these regulations will improve women’s health in the form of internal regulations (PhD in health promotion, occupational health promotion specialist.

The topics related to women’s health need to be very visible, clear, and transparent in the hospital’s policy. When developing the strategic direction for the hospital, as well as operational planning, women’s health needs to be considered as a core factor to inform managers and leaders about the required actions. As participants reported, the current hospital policies and practices require updating and adjustment concerning women’s health to truly meet the goals.

“Well, we can say that in Adelaide’s statement, this issue has been mentioned exactly. In the workplace, at least if it is not separate in the policy, health issues and now women’s issues should be mentioned in the policy… (workplace health promotion specialist).

a systemic view [is required] and these differences should be taken into account. Otherwise, you cannot have a written program for your collection, and this must exist in hospital processes… (health promotion specialist, university professor).

### Recognition through career development

In this study, human resource development emphasized the problems and issues faced by women, including meritocracy, respecting women’s rights, and human resource management. Communicative competence and achieving meritocracy in the workplace were among the factors that the participants reported. Managers and leaders who are informed and understand women’s issues can assist with creating a more personalized approach, addressing each case individually, so that women can thrive personally and professionally in their careers. Creating a healthy workplace for women in hospitals results in a win-win situation for both female employees and the organization

If we can have a representative in the workplace who advocates for women’s issues and challenges, this can significantly impact the [work] effectiveness of working women (A 47-year-old specialist in nephrology with 9 years of experience).

When I want to retire, if my husband does not support me, I have nothing of my own. I came here when I was 24 years old, and I have been working here for 24 years. But I don’t have anything for myself (48-year-old nurse).

Some of the key drivers of satisfaction in female employees reported include respecting women’s rights, equal job opportunities, and at the same time, equitable allocation of resources, acknowledging women’s specific situations, respecting women’s positions, and not underestimating women’s capacities in leadership and management positions. It has been stated that these measures are effective in enhancing women’s satisfaction, performance, and well-being in workplaces. The existence of patriarchal hierarchy and male-dominated structures leads to discrimination between men and women, resulting in unequal job opportunities, with most men being placed in management positions. However, the inclusion of women in management roles can benefit both the system and the understanding of the challenges faced by working women, facilitating their resolution.

Unfortunately, there are mostly male structures in our hospitals, I say that these should be broken areas for growth should be created, and also equal job opportunities should be given to women. (Health promotion specialist, professor of education and health promotion, university faculty member)

Job security and the sense of financial independence profoundly influence the overall sense of security women derive from their workplace. Numerous participants reported that the presence of discrimination in allocating financial resources to women significantly affects their sense of security and financial independence. Ensuring that the hospital and its management system address this aspect of women’s needs can play a crucial role in establishing an effective workplace conducive to women’s health.

If I want to say about job security if you are an official, you have more job security, but if you are a company, you can be fired at the beginning of the year, so there is practically no job security. (MS, head of CCU department, 23 years of experience

### Empowerment through systematic networking

Hospital managers and leaders aiming to prioritize the empowerment of women in various fields can achieve greater success in creating healthy work environments for women. Developing a safe atmosphere with empowering approaches to assist female employees in thriving personally and professionally is essential for enhancing performance. Examples from participants include improving communication skills, enhancing self-confidence, and developing control and self-efficacy to navigate situations for fulfillment. Different stages of their careers need to be taken into account, and women should be mentored and supported to achieve their goals for a meaningful and fruitful outcome, as they progress into their careers. Involving women in hospital health promotion programs and establishing women’s health self-help groups can also contribute significantly to the creation of a healthy work environment for hospital managers.

More than all of this, you can work in the field of empowerment, and increase their courage., This will help them grow in the system (Mr. Health Promotion Specialist, Professor of Education and Health Promotion).

Many times, there is no need to physically assist this lady; perhaps if there is someone who listens to her concerns, 90% of her problems would be solved. The ability and social support they receive from their colleagues or managers help them to be more successful in areas such as childcare, marital life, or even in their profession. This can happen through the support received in colleague groups or counseling sessions provided by their colleagues. (A charge nurse in CCU with 23 years of experience)

### Psychological safe environment for women’s health

The category psychologically safe environment for women’s health included fostering satisfaction, security, and joy at work, and striking a balance in work-life harmony.

### Fostering satisfaction, security, and joy at work

Women emphasized that elevating the perceived psychological safety within the workplace not only boosts their confidence but also empowers them to operate and offer services in a more serene environment, alleviating additional stress and perceived psychological pressure. Moreover, factors such as the constructive handling of mistakes and errors at work, managers’ comprehension of women’s work and family conditions, and the establishment of career incentives and encouragement for managers to motivate women emerged as pivotal elements influencing their perceived satisfaction and psychological security, as highlighted by the participants.

The patient is seeking a service, and any negligence or mistakes should not be attributed to them. Accountability lies with the healthcare provider, particularly the nurse in question. However, the crucial focus should be on addressing concerns in a healthy and stress-free environment. In my opinion, an informative environment fosters better understanding and resolution (female, 48 years old, supervisor, 24 years of work experience).

Employees, especially women, should be motivated to work. Be encouraged for the positive work you do one day, even the simplest work, when you are encouraged, you will find motivation and you will do it many times in the future. (Female, 28 years old, nursing expert with 3 years of work experience)

Several participants highlighted that a serene atmosphere, free from additional tensions, along with an aesthetically pleasing and fresh workplace, coupled with the provision of necessary equipment for a favorable work environment, could significantly contribute to relaxation and enhance their working conditions. They noted that therapeutic work environments, such as hospitals, may inherently introduce various stresses, and additional tensions could adversely impact their health, particularly their mental well-being. Moreover, an appealing physical work environment was emphasized as a factor that can alleviate tension.

The first thing is to be calm in the work environment. When your environment is calm, your mental health is ensured and you can think and make decisions better. (Head of the women’s ward, 40 years old, 15 years of work experience)

For example, a small change in the women’s resting place will improve their mood. Every once in a while, if they buy a flower for their workplace, or make a small change in the space, it might have an effect and change their mood, for sure.

The hospital managers can sometimes make a happy program and gather the staff, for example, a happy program will be held in the amphitheater hall (29-year-old lady, in charge of education, 5 years of work experience).

### Striking the balance in work-life harmony

The participants asserted that achieving work-life balance for women stands as one of the most crucial factors impacting all facets of women’s lives, encompassing both work and family, particularly for those employed in a hospital setting. The myriad roles that women undertake within the family, society, and workplace can often clash, leading to challenges. Establishing a harmonious equilibrium between work and life necessitates support from various sources, including hospital managers and families. Simultaneously, many women contend with the conflicting pressures of different roles, highlighting the imperative need for training and empowerment.

According to the participants, the hospital’s support for women’s family conditions emerged as one of the paramount contributors to work-life balance. Notably, accommodating features such as flexibility in time and workload for pregnant women can prove instrumental in mitigating risks associated with work pressure and societal roles.

A woman who is pregnant surely has problems, and she cannot easily do all the work with a lot of pressure; this pressure will harm her. The system can work more flexibly for pregnant women.

On the other hand, family problems faced by personnel, such as taking care of children and babies under one-year-old, mothers dealing with children with special needs, and managing their care, were also reported by many participants.

When the hospital provides conditions and facilities for women to take care of children, obviously women can come to work more easily, and they can take care of their problems at home more easily (female, 28 years old, a nursing expert with 4 years of work experience).

Numerous women employed in hospital settings encounter challenges, such as reservations about accepting night shifts or working on holidays, particularly when it conflicts with their partner’s schedules. Some participants highlighted that night shifts and holiday work can disrupt women’s family lives. However, participants also noted that with the cooperation and support of managers and officials, including department heads and supervisors, in arranging their work schedules until these family challenges find suitable resolutions, a balanced and tension-free environment can be established. Additionally, the impact of such support on their work-life balance was deemed significant.

The people who work here are married, many of them have partners who are not nurses and have other jobs. Therefore, it is difficult for them if their spouses work at night or on holidays, and management by being aware of these problems and creating a supportive environment in these conditions can reduce many of these problems. (Ms., CCU supervisor 45 years old, 20 years of work experience)

### Thriving for positive social connections supporting women’s health

This category included two key sub-categories:

1. Fostering solidarity and social cohesion
2. Socially secure environment for women

Fostering Solidarity and Cohesion encompass the development of positive employee interactions, positive working relationships, and the cultivation of social support among colleagues. Participants emphasized that non-discrimination in employees’ tasks, effective organizational management, collaborative problem-solving within departments, mutual respect among employees, and the establishment of efficient horizontal communication were crucial factors contributing to professional solidarity and cohesion. Fair organizational promotions, devoid of informal relationships and adhering to established rules, fostered an environment where there was no perceived difference between the work of female employees and that of all individuals, eliminating discrimination. Positive interactions between department managers, employees, and women, coupled with effective communication and mutual respect among colleagues, created a conducive atmosphere for women to find peace in an environment typically fraught with stress and tension within the hospital.

Career promotion is very important here. In a country like Japan, your colleague tells you to give me your hand so that I can take you higher, so I go one step higher. But this is not the case here, either they push you down or they are so pessimistic towards each other that if they are on the same level, they are still jealous of each other (38-year-old female, nurse expert, head of NICU department).

I like it very much, when there is a problem between me and my colleague, we solve it ourselves and it is not taken to the office of the director or nurse, because our job has enough margins (26-year-old woman, nursing expert with 4 years of work experience).

The manager is like the mother of the family, he can make the atmosphere very good, he can calm the atmosphere with the management he does, and he can make the children all get along well. (Female, 47 years old, nursing expert with 24 years of work experience)

Creating a Socially Secure Environment for Women is identified as one of the most pivotal factors contributing to a healthy workplace and promoting women’s health. Participants regarded it as the foremost factor in ensuring the safety of women in the workplace, encompassing both physical and psychological dimensions. This includes the prevention of horizontal violence, organizational coercion, and violence perceived by patients and companions, as well as the provision of both social and economic security.

An unreasonably high volume of work, compulsory shifts, placement in different hospital departments without choice, inadequate compensation for substantial workloads, an unsuitable work environment, workplace stressors, and a lack of facilities constitute factors categorized as organizational coercion (vertical violence). This phenomenon involves staff and nurses and is observable by managers. While instances of harassment might not always garner sufficient attention, participants highlighted that these organizational challenges significantly impact the overall quality of work life for these employees.

Unfortunately, there is a heavy workload that when you go home, you are tired and you carry all the anger and violence that you saw at work home…” (female, 28 years old, a nursing expert with 3 years of work experience)

In our work, overtime is compulsory, I don’t want it, but our job is compulsory and I have to come to work every hour. (Nursing expert lady, 42 years old, 18 years of work experience)

Violence inflicted by patients and their companions, whether physical or verbal, presents a significant challenge. Factors such as a lack of facilities and power imbalances can contribute to instances of verbal violence between colleagues or physical altercations between patients and their companions. The repercussions of such violence can be severe, resulting in a decline in service quality, patient discomfort, and, in extreme cases, even patient harm or death. This poses a considerable risk to nurses, doctors, paramedics, and hospital staff. Among the mentioned concerns. The presence of security in working relationships with male colleagues emerged as one of the most crucial factors contributing to the creation of a healthy workplace for women, as reported by the study participants.

We have cases where as soon as the patient comes in, he breaks the glass, throws files, throws anything he can get & (MS. Supervisor, 48 years old, 24 years of work experience)

The companion is very agitated and angry, and they curse the patient for not being stable. She sees her patient’s family dying and is angry and vents all her anger on the female nurses who have no support…” (MS, nursing expert, 38 years old, shift manager, 13 years of work experience).

Women also highlighted that the imrporatance of interpersonal dynamics, and how important it is for men to exhibit awareness and discretion in their communication and behavior. For example, jokes and conversation style should be approached with consideration, recognizing that what may be acceptable among male peers might not be suitable when interacting with women. It underscores the need for a discerning choice of words and highlights the importance of being mindful of physical contact. Thic can result in creating a respectful and inclusive environment, ensuring that interactions are thoughtful and well-received across diverse social contexts

Men joke among themselves. No one indeed has a purpose, for example, men should be aware of the style of jokes they make or the style of their conversation, the words that are used when talking to each other are not used when talking to women, they should be aware of physical contact, this All kinds of things are influential. (Female, 28 years old, radiology expert, 3 years of work experience).

### Advancing holistic health for women

The category advancing holistic health from women highlights the attention to holistic care and not only on the physical aspect of health but also the psycho-social and health promotion aspects. This included Occupational health and safety, Promoting healthy lifestyle behaviours, and the Welfare of women.

### Occupational health and safety

Paying attention to ergonomics tailored to the women’s work environment, ensuring workplace safety aligned with the characteristics of women in the hospital, and providing a health-conscious workplace are critical factors, as reported by participants. Compliance with load-carrying regulations for women, furnishing equipment tailored to the ergonomics of women, and considering equipment suitable for the structure of the male body were also emphasized. Furthermore, women stressed the need for special attention to the musculoskeletal system of women, given its differences from that of men. Particularly, during pregnancy, and considering the physical strain from patient transportation that women experience in environments such as hospitals, attention to musculoskeletal health becomes paramount for maintaining their physical well-being. Women emphasized the necessity for specific planning and considerations at the management and leadership levels to create a healthy workplace in hospitals. This not only impacts their overall well-being but also influences their performance.

Because we work at the patient’s bedside, the physical pressure is higher here. Standing for long periods during visits, giving medicine, and moving patients should be taken into account (female, 40 years old, head of the women’s ward, 15 years of work experience)

But our environments generally do not respect women’s indicators, in the sense that even if the matter is examined from the point of view of ergonomics, it is examined as a total, that is, the ergonomic conditions specific to women are not examined. (MS. member of the board of directors and adviser to the director in family and women’s affairs, member of the women’s working group in the field of the Ministry of Petroleum)

Understanding the physical and physiological differences between genders underscores the importance of proactively addressing health and safety concerns. This proactive approach not only ensures immediate well-being but also establishes a sustainable framework for long-term workplace safety. For example, ensuring the safety of the work environment tailored to the characteristics of women in the hospital was identified as a crucial factor contributing to the creation of a workplace that promotes women’s health, as emphasized by the participants.

For example, women are more sensitive to electrocution. In a certain amp, where men don’t get electrocuted, women get electrocuted. Because their body resistance is less. It means that they are more communicative than men. Women have more body water and are more affected by electrocution, and this should be taken into account by the system. (Male, PhD. in Occupational Health, Deputy of the Occupational Health Center of the Ministry of Health)

Understanding the physical and physiological differences between genders underscores the importance of proactively addressing health and safety concerns. This proactive approach not only ensures immediate well-being but also establishes a sustainable framework for long-term workplace safety. For example, chemical exposures and physical elements, including light and sound, may impact women differently due to physiological and physical distinctions between genders. It is, therefore, essential to address these workplace factors, ensuring proper ventilation and controlling heating and cooling conditions to meet the specific needs of working women. Participants emphasized the significance of paying attention to air conditioning and temperature regulation to create favorable working environments for women experiencing various reproductive phases such as menstruation, pregnancy, and menopause. Hence, careful attention to these factors holds special importance in establishing a healthy workplace for women.

The hospital must provide the conditions for the mother so that neither the mother nor the baby that is going to be born is harmed, especially in departments such as CT scans or radiology that are in contact with radiation… (Female, Bachelor of Occupational Health, 27 years old, 3 years of work experience)

The most important thing and we do not have it in this department, is proper ventilation for the health of the space, and the fact that women’s body temperature rises during one month due to hormonal changes, and they tolerate this space without proper ventilation or improper heating system. It’s really hard and suffocating. (Lady, 28- years- old assistant, 5 years of work experience)

### Promoting healthy lifestyle behaviours

It is crucial to consider holistic lifestyle behavior programs and approaches to enhance women’s care. This encompasses women’s periodic tests and examinations, promoting healthy lifestyle behaviors such as physical activity, and healthy eating. Specializing in women’s periodic tests and examinations, with a focus on the genitourinary system and the unique conditions women experience during different periods of fertility, emerged as crucial factors influencing women’s health, as reported by study participants. Many of the periodic care practices in workplaces are often general and not specifically tailored to the unique needs of women.

Women’s issues are specific matters and should be given attention. For example, in the chest examination section, issues related to breasts and such are not distinctly specified. The physician simply marks the breast section and moves on, without conducting a specific examination for women’s issues. ” ”

Women highlighted opportunities for physical activity in hospitals as a pivotal factor contributing to the establishment of a health-promoting workplace. For example, creating a supportive environment in workplaces, such as providing breaks, addressing constraints like financial or time limitations, and determining suitable times for utilizing facilities were identified as crucial elements that could significantly impact the creation of a healthy environment for women in the workplace.

I say let’s set some hours so that we can exercise. We have morning, evening, and night shifts for our work, clubs should also have morning, evening, and night shifts, for example, until 8:00 PM, and a woman can go to exercise after 8:00 PM. Sports themselves solve many of the children’s problems, both mentally and physically, and it affect their health in every way. (Female, 44 years old, head of the neonatal ward, 15 years of work experience)

In addition, numerous participants emphasized that hospital policies promoting proper nutrition could significantly contribute to enhancing the health of employees, especially women with high workloads in hospitals. Women working night shifts, extended hours, pregnant or lactating, and those menstruating may experience compromised health and reduced fertility and efficiency if not provided with healthy nutrition at the appropriate times. Moreover, many participants highlighted the lack of sufficient time for meals as one of the major challenges, representing a significant obstacle to maintaining a healthy diet.

In my opinion, nutrition is very important, not much attention is paid to this issue, but it is really important for women, especially those who are breastfeeding, to be able to eat on time and to be given proper food… (MS., 45-year-old office manager, 18 years of the record)

### Welfare of women

Participants highlighted support and facilities, including health-related amenities and welfare for staff families, such as childcare facilities. A crucial factor contributing to creating a healthy workplace and promoting women’s health revolves around the concerns that women harbor for their children, their care, and the environment in which they are looked after.

A child’s coach is very important. In the hospital where my sister works, the teacher beats the child. Well, it is very important not to disturb the child. My sister was getting nervous, she couldn’t work at all and this made her change the coach. she was so stressed at work; that she visited the child every few minutes to ensure that nothing had happened to the child. (Ms., assistant, 28 years old, 3 years of work experience)

The amenities and facilities that the hospital provides to women, tailored to their working hours, along with provisions for resting within the hospital during extended shifts, can assist in maintaining a healthy work-life balance and fostering a serene work environment. For instance, shopping stores designed to align with the working hours of women employed in the hospital can prove beneficial for their well-being.

It is better if there are stores with hours that match our working hours. We have a store now, but the time is not such that we can use it, I can’t go shopping there in the middle of the day, it’s great when I’m done with work, I can go shopping for my home. (Female, 38 years old, NICU department manager, 15 years of work experience)

There is no proper resting room for nursing personnel at all. The worst rooms are usually given for employees to rest (MS., 45 years old, 15 years of work experience)

Offering facilities outside the hospital environment, including sports and recreational amenities, can provide women with a means to distance themselves from the demands of hospital work. Aligned with their life schedules, women can utilize external facilities such as stores, swimming pools, and sports facilities that have been arranged for them in coordination with the hospital.

The employee wellness or recreation centers should be indoors, because women don’t have time to go outside to do this work, or now they can do it for half an hour, after their work hours or half an hour, in the middle. When they have free time, they can go there and do a group exercise, it will be much better if they can do this (40-year-old woman, nursing expert, 18 years of work experience

## Discussion

This study focused on the key drivers of health and well-being in women working in healthcare settings, such as hospitals, undertaking various roles ranging from allied health positions to clinical and managerial roles [23]. Women are driving the healthcare industry as they constitute the main employees across various departments in healthcare settings [24]. Therefore, adopting a comprehensive approach to workplace health and well-being for women is imperative. This encompasses a broad spectrum of health-related factors that can impact their health, well-being, and productivity. Hence, it is necessary to tailor the working environment based on their needs and preferences, addressing both physical and psychosocial aspects of health and well-being. This approach also considers the establishment of work strategies, structures, culture, and planning that support health-promoting choices [25]. In this regard, the WHO has formulated a framework and pertinent guidelines for a ‘healthy work environment,’ emphasizing a holistic perspective on workplace health [3] In this comprehensive, evidence-based model, health promotion programs in the workplace have been optimally developed [25].

Leaders’ and managers’ support in the healthcare system is the foundation for creating a work environment that promotes women’s health. As highlighted in the second health promotion conference in Adelaide in 1988, women are the primary health promoters globally, requiring more attention to their health and well-being. The statement also emphasizes that the needs and priorities of women must be taken into consideration when formulating policies and support mechanisms for caregiving activities, including support for mothers with children, maternity leave, and leave for individuals under the guardianship of women. These considerations should be incorporated into the agendas of policymakers at different levels [26]. Hence, the differences across genders need to be taken into consideration when looking at women’s health and well-being to provide tailored services suited for women [27].

This study showed that one of the important factors having a significant impact on promoting women’s health in workplaces was their active participation in the development of programs and policies related to women’s health. The presence of women in managerial positions is crucial for creating a healthy workplace, and their cooperation and participation in formulating policies and programs related to occupational health and safety can help managers understand the issues and problems faced by women. Additionally, representatives from all groups, especially gender groups, should be present in health and safety committees to ensure a healthy workplace for both sexes [20]. Incorporating women’s health into the vision and policy statements of various organizations and workplaces can help create healthy environments, particularly for settings such as healthcare that are dominated by women [11, 28].

Discrimination in job opportunities and wages for women is another factor that should be considered in human resource management. Women constitute half of the human capital in society and their true contribution can make transformational changes [29]. Social factors play a role in shaping the gender distribution within the healthcare workforce. There is a tendency for women to be more prevalent in roles that are often undervalued and lower in status, such as nurses, midwives, and healthcare assistants. While, men are frequently observed in positions with higher ranks and earnings, such as doctors and specialists. This pattern underscores the need for ongoing efforts to address gender imbalances and promote greater equity within the healthcare sector [30]. Lucero Soledad’s study suggested that perceived discrimination can impact women’s health and well-being adversely [31, 32]. Stamarski’s study also found that organizational structures, processes, and practices, such as leadership, structure, strategy, culture, and human resource policy, were interrelated and might contribute to discrimination [33]. Negative workplace experiences, including gender discrimination, predict decreased job satisfaction and increased burnout in female employees [33]. Workplace gender discrimination, particularly in wage allocation, can jeopardize women’s mental health, leading to increased risks of depression and stress [33].

Two interconnected categories of psychological and social aspects of health indicated the importance of psycho-social safe environments where women feel heard and belong. Creating supportive work environments for women, especially those working in settings such as hospitals that entail significant work pressure and workload, can be one of the important policies for organizations and hospital work environments [20]. Working in medical centers can be exhausting. Dealing with patients who have lost their health and morale is very difficult and taxing, causing anxiety and concern for the staff in medical centers [34]. Making the necessary plans by the hospital and managers to mitigate women’s stress and job burnout, and planning to create a balance between women’s work and life, as well as establishing a workplace that promotes women’s health, is crucial [35]. Chou et al.‘s study reported that long-term exposure to job-related stress led to burnout, which can be defined as a psychological syndrome that may occur when employees are faced with a stressful, demanding workplace. The combination of high occupational demands and low resources can endanger their health [36]. However, it should be noted that facing stress in workplaces, especially in hospitals, is unavoidable, and eliminating stress is not possible. Hence, learning coping strategies, enhancing resilience [37], and incorporating recreational programs are beneficial for dealing with stressful situations [38]. To enhance well-being, implement empowering programs and interventions that focus on promoting healthy lifestyle behaviors, including physical activity, meditation, training courses, mental health counseling tailored for women, and psychological group meetings. The study Studies suggested adapting and controlling psychological pressures on women [39, 40], including group therapy training sessions to identify stressful situations in the work environment and provide them with the necessary coping tools/strategies [41].

Work and family are essential social institutions, and addressing role conflicts for women, especially those in the healthcare sector like nurses, doctors, and paramedics, is crucial for achieving a work-life balance [42]. Pregnant, breastfeeding, and women facing unique family challenges were reported issues. Addressing these concerns contributes to creating a psychologically healthy workplace. Studies highlight that role conflicts have a profound impact on women, burdening them with multiple tasks and potential work-family conflicts. Resolving these issues is crucial, as the consequences extend beyond the individual, affecting both the organization and, on a broader scale, society [43]. Pandey’s study found that achieving work-life balance is a challenge that can be addressed through effective management strategies [44]. Namdari et al.‘s study reported that high work-family conflict might have negative consequences, such as job and life dissatisfaction, significant worry, psychological pressure, physical symptoms, depression, job burnout, and marital dissatisfaction, as well as reducing accuracy and quality in providing services [45]. Polat et al.‘s study also acknowledged that with support from the organization and family, gaining experience during working years, and through training and empowerment, can be effective in addressing this issue [46]. One of the factors that can contribute to establishing a work-life balance for women is the support that the workplace and managers can provide to female employees in case of family problems, such as having infants or sick children, family, and marital problems. Workplace support in case of family problems could be considered as one of the important factors affecting women’s work-life balance [47]. For example, the effect of role conflict on anxiety can be moderated by social support, appropriate resources [48], psycho-social trainings related to the quality of work life, resilience skills, and managing stress and burnout [49]. A supportive organizational climate, characterized by collaborative management, positive feedback mechanisms, and workplace control, enhances managerial effectiveness. Such an environment facilitates managers and clinical consultants in fulfilling their roles, supporting staff effectively, and fostering commitment, teamwork, and overall well-being among participants [50]. The safety of women in the hospital is one of the most important factors that can contribute to creating healthy workplaces for them, as the results showed, to reduce the amount of violence in the workplace, especially in the hospital, managers must provide opportunities to decrease violence rate in the hospital and reduce the barriers to reporting these cases [51].

Tailoring the workplace to meet women’s needs is vital for their well-being and performance. Despite the integration of men’s and women’s jobs in hospitals, experts argue that distinct work statuses, time allocations, and priorities persist. Studies on altering gender composition in physically demanding jobs emphasize the significance of gender-specific ergonomics, considering differences in strength, anthropometry, and their relation to equipment and workplace design. The lack of attention from managers and policymakers to women’s ergonomic issues may be influenced by these factors [52]. In addition, focusing on activities promoting a healthy lifestyle and offering health promotion interventions, such as physical activity and nutrition programs, for female hospital employees necessitates specialized policies. Several working women recommend management adjustments, such as dedicated time for physical activity or accessible facilities, to integrate it into their schedules. Fatemeh Bakhtari et al.‘s study indicates that the social environment, with both positive and negative aspects, and self-efficacy directly and indirectly influence physical activity among working women. This research also highlights that individual factors, the social environment, and the physical environment directly impact all forms of physical activity [51]. Furthermore, addressing workplace obstacles impacting women’s health and physical activity, such as time constraints, inadequate social support, restricted access to sports facilities, and associated costs, is essential [53]. The critical organizational factors requiring attention to support women’s health and well-being include limited work flexibility, inadequate team leader support, and scheduling training sessions outside regular working hours. These can be barriers to women’s active participation in workplace health promotion programs. The study recommends that health service providers should in China receive support from team management for programs aimed at enhancing the health of female employees. Strategic planning of work shifts is also suggested to facilitate the participation of all individuals involved [54].

### Strengths and limitations

This study is a unique research on women’s well-being in health-related settings by gathering data directly from a wide range of participants in hospitals. However, there were also some limitations. The research was conducted at only one hospital in Tehran, and generalization needs to happen with caution. To mitigate this limitation, we aimed to include maximum diversity in terms of job types, education levels, and workplace departments. In addition, there were issues with participation in the study due to busyness and heavy workloads, especially in the clinical departments, which were addressed through prior arrangements and scheduling interviews at mutually convenient times.

## Conclusion

Establishing a healthy and safe work environment for women in healthcare is crucial for their performance, satisfaction, and overall well-being. The unique conditions of hospitals, characterized by high customer volumes, substantial workloads, and extended shifts, make it imperative to prioritize the creation of a healthy workplace, especially considering that women play a central role in healthcare sector. Ensuring a healthy workplace for women necessitates a holistic approach, addressing various aspects of health and well-being that align with their needs and preferences. This includes fostering a healthy mental and social environment, ensuring well-being across physical, psychological, and social dimensions through active participation, and providing health promotion services tailored to women working in the hospital.

## Data Availability

Data used in this study is analyzed and the data is available from the first author upon reasonable request.

## Abbreviations

WHO: World Health Organization
MAXQDA: a software program designed for computer-assisted qualitative and mixed methods data, text, and multimedia analysis in academic, scientific, and business institutions
